# Nox2 Oxidase Activity Accounts for the Oxidative Stress and Vasomotor Dysfunction in Mouse Cerebral Arteries following Ischemic Stroke

**DOI:** 10.1371/journal.pone.0028393

**Published:** 2011-12-02

**Authors:** T. Michael De Silva, Vanessa H. Brait, Grant R. Drummond, Christopher G. Sobey, Alyson A. Miller

**Affiliations:** Department of Pharmacology, Monash University, Clayton, Victoria, Australia; Julius-Maximilians-Universität Würzburg, Germany

## Abstract

**Background and Purpose:**

Post-ischemic oxidative stress and vasomotor dysfunction in cerebral arteries may increase the likelihood of cognitive impairment and secondary stroke. However, the underlying mechanisms of post-stroke vascular abnormalities, as distinct from those causing primary brain injury, are poorly understood. We tested whether augmented superoxide-dependent dysfunction occurs in the mouse cerebral circulation following ischemia-reperfusion, and evaluated the role of Nox2 oxidase.

**Methods:**

Cerebral ischemia was induced in male C57Bl6/J wild-type (WT) and Nox2-deficient (Nox2^-/-^) mice by middle cerebral artery occlusion (MCAO; 0.5 h), followed by reperfusion (23.5 h). Superoxide production by MCA was measured by L-012-enhanced chemiluminescence. Nitric oxide (NO) function was assessed in cannulated and pressurized MCA via the vasoconstrictor response to *N* ^ω^-nitro-L-arginine methyl ester (L-NAME; 100 µmol/L). Expression of Nox2, the nitration marker 3-nitrotyrosine, and leukocyte marker CD45 was assessed in cerebral arteries by Western blotting.

**Results:**

Following ischemia-reperfusion, superoxide production was markedly increased in the MCA of WT, but not Nox2^-/-^ mice. In WT mice, L-NAME-induced constriction was reduced by ∼50% in ischemic MCA, whereas ischemia-reperfusion had no effect on responses to L-NAME in vessels from Nox2^-/-^ mice. In ischemic MCA from WT mice, expression of Nox2 and 3-nitrotyrosine were ∼1.4-fold higher than in the contralateral MCA, or in ischemic or contralateral vessels from Nox2^-/-^ mice. Vascular CD45 levels were unchanged by ischemia-reperfusion.

**Conclusions:**

Excessive superoxide production, impaired NO function and nitrosative stress occur in mouse cerebral arteries after ischemia-reperfusion. These abnormalities appear to be exclusively due to increased activity of vascular Nox2 oxidase.

## Introduction

Cerebrovascular dysfunction, consisting of deficits in nitric oxide (NO)-dependent endothelial function and vasodilatation, occurs early following cerebral ischemia-reperfusion [1]. These vascular abnormalities may then limit brain perfusion and accelerate inflammation and death of neuronal tissue within the compromised but potentially salvageable penumbra, thereby increasing the risk of secondary stroke and cognitive impairment [2], [3]. Despite progress in understanding primary mechanisms of neuronal cell death during ischemia [4], translation of that knowledge into effective stroke therapies has so far been unsuccessful [5]. Consequently, an increased focus on targeting key vascular mechanisms for improving stroke outcome has been advocated [6].

Oxidative stress, characterized by excessive levels of reactive oxygen species (ROS) such as superoxide and hydrogen peroxide, is a major cause of neuronal injury after cerebral ischemia-reperfusion [6]. ROS levels are elevated in the cerebral vasculature during reperfusion [7], [8], [9], [10], [11], and are suspected to be an underlying cause of post-ischemic endothelial dysfunction [9], [10], however, their enzymatic source(s) is yet to be defined. The NADPH oxidases are the only enzymes yet discovered with the primary function of generating superoxide [12], and they are major sources of ROS in the cerebral vasculature under physiological conditions [13]. This family of enzymes comprises two membrane-bound subunits, including a Nox catalytic subunit and p22phox, as well as different combinations of several cytoplasmic subunits [12], [13]. In cerebral blood vessels, at least three isoforms of NADPH oxidase are expressed, namely Nox1-, Nox2-, and Nox4-containing NADPH oxidases (or ‘Nox oxidases’) [12], [13]. The Nox2 oxidase is predominantly expressed in the endothelial cell layer of cerebral arteries, and this isoform is emerging as a major source of pathological ROS in cerebral vessels [14], [15], [16], [17]. Although experimental studies have demonstrated a causative role for Nox2 oxidase in neuronal [18], [19], [20] and blood-brain barrier damage [21] after ischemic stroke, it is unclear whether Nox2 oxidase contributes to increased superoxide levels and/or endothelial dysfunction in the cerebral circulation following ischemia-reperfusion. Currently available pharmacological inhibitors of Nox oxidases have limited utility for defining molecular pathways due to their lack of isoform selectivity and/or their off-target effects [12], and so definitive evidence for a causative role for any of these enzymes requires the use of genetically modified mouse models. Therefore, the aim of the present study was to firstly test whether augmented superoxide production and endothelial dysfunction occur in the mouse cerebral circulation following ischemia-reperfusion, and secondly to evaluate the role of Nox2 oxidase in these effects using Nox2-deficient mice.

## Materials and Methods

All procedures were approved by the institutional animal ethics committee. In total, 72 male C57Bl6/J wild type (WT, 25.9±0.3 g) and 24 male Nox2-deficient (Nox2^-/-^: 27.3±0.4 g) mice were studied. Nox2^-/-^ mice were originally generated in the laboratory of Prof. Mary Dinauer [22] and bred at Mouseworks (Clayton, Australia). Nox2^-/-^ mice were backcrossed to the C57Bl6/J strain for at least 10 generations. Mice were studied at 8 to 12 weeks of age and killed by inhalation of isoflurane followed by decapitation. In all, 15 WT and 6 Nox2^-/-^ mice were excluded from the study which occurred when, during the surgical procedure to induce focal cerebral ischemia-reperfusion: (1) there was inadequate (<70%) reduction in regional cerebral blood flow (rCBF) (n = 1 for WT and n = 1 for Nox2^-/-^); or (2) technical or anaesthesia complications arose during surgery (n = 14 for WT and n = 5 for Nox2^-/-^).

### Focal cerebral ischemia-reperfusion

Mice were anesthetized with a mixture of ketamine (80 mg/kg, i.p.) and xylazine (10 mg/kg, i.p.). Body temperature was maintained at 37.5°C with a heat lamp throughout the procedure and until animals regained consciousness. Focal cerebral ischemia-reperfusion was performed in mice by transient intraluminal filament-induced middle cerebral artery occlusion (MCAO) as previously described [18], [20], [23]. rCBF in the area of the cortex supplied by the MCA (approximately 2 mm posterior and 5 mm lateral to bregma) was monitored and recorded prior to the induction of cerebral ischemia and for the first 30 min of reperfusion using trans-cranial laser-Doppler flowmetry. For sham MCAO surgery, the common carotid artery was visualized, but the MCA was not occluded.

### Evaluation of neurological function, cerebral infarct and edema volume

At the end of the experiment (24 h), neurological function was evaluated by an observer blinded to the genotype using the hanging wire test [18], [20], [23]. Briefly, mice were suspended from a wire 30 cm high for up to 60 s and the average hanging time (i.e. latency to fall) of 3 trials with 5 min rest in between was recorded. Cerebral infarct and edema volumes at 24 h were also evaluated as previously described [18], [20], [23]. Briefly, brains were sectioned (30 µm coronal sections; 420 µm apart) using a Leica CM1850 cryostat and thaw mounted onto 0.1% poly-L-lysine coated slides. Sections were immersed in 0.1% thionin (2 min), rinsed with distilled H_2_O, then immersed in 70% ethanol (2 min) followed by 100% ethanol (2 min). Sections were then dipped in xylene and cover slipped with DPX mounting media. Thionin-stained sections were imaged with a CCD camera (Cohu Inc., San Diego, California, USA) mounted above a light box (Biotec-Fischer Colour Control 5000, Reiskirchin, Germany). Total infarct volume was quantified using ImageJ image analysis software (Version 1.42q, NIH), correcting for brain edema, according to the following formula: CIV  =  [RIA – (RHA – LHA)] × thickness of slice (CIV, corrected infarct volume; RIA, right hemisphere infarct area; RHA, right hemisphere area; LHA, left hemisphere area). Edema volume was estimated according to the formula: EV  =  (RHA – LHA) × thickness of slice (EV, Edema volume). Infarct and edema volumes for all sections were totaled and expressed as mm^3^.

### Measurement of superoxide production by middle cerebral arteries

MCA were isolated from the contralateral (non-ischemic) and ischemic cerebral hemispheres of WT and Nox2^-/-^ mice after 23.5 h reperfusion. In addition, MCA from left and right hemispheres of sham-operated WT mice were isolated. Briefly, using a dissection microscope, the MCA was located and then dissected free of the brain, starting ∼1–2 mm distal to its origin at the Circle of Willis and finishing at the end of its second branch. Basal and phorbol-12, 13-dibutyrate (10 µmol/L; PDB: Nox2 activator)-stimulated superoxide production was measured consecutively in the same MCA using 100 µmol/L L-012-enhanced chemiluminescence and expressed in counts/mg of dry tissue weight, as previously described [14], [16], [24].

### Assessment of nitric oxide function in middle cerebral arteries

MCA were isolated from the ischemic and non-ischemic hemispheres of WT and Nox2^-/-^ mice after 23.5 h reperfusion, and were mounted between two microcannulae in a pressure myograph (Living Systems Instrumentation Inc.). Arteries were superfused with warm (37°C) carbogen-bubbled (95% O_2_, 5% CO_2_) Krebs-bicarbonate solution (composition in mmol/L; NaCl 118, KCl 4.5, MgSO_4_ 0.45, KH_2_PO_4_ 1.03, NaHCO_3_ 25, glucose 11.1, CaCl_2_ 2.5). Intraluminal pressure was gradually increased to 60 mmHg and maintained at this level with a pressure servo unit without further intraluminal perfusion. Each MCA was allowed to equilibrate for 15 min and baseline diameter was then measured. Functional experiments were performed on both ischemic and non-ischemic MCA in parallel, and arteries from a WT and a Nox2^-/-^ mouse were typically studied on the same day.

NO function was assessed via the constrictor response to the NO synthase (NOS) inhibitor, L-NAME (100 µmol/L) [16], [24], [25], [26]. Constrictor responses were recorded when a steady level in diameter was reached (after ∼30 min). Cumulative concentration-dependent constrictor responses to either the α_1_-adrenoceptor agonist phenylephrine (1–1000 nmol/L) or the thromboxane A_2_ mimetic U46619 (1–1000 nmol/L) were also assessed.

### Western Blotting

Ischemic and contralateral MCA were isolated from WT and Nox2^-/-^ mice 23.5 h after reperfusion. Protein expression levels of Nox2, the nitration marker 3-nitrotyrosine, endothelial NOS (eNOS) and the pan leukocyte marker CD45 were measured in MCA homogenates using Western blotting as previously described [14], [16], [27]. For Nox2, 3-nitrotyrosine and eNOS, ischemic or contralateral MCA from two mice were pooled for each experiment. For CD45, spleen homogenates were used as a positive control. In preliminary experiments, we determined that 30 µg of protein was required to detect CD45 immunoreactivity (>150 kDa) in spleen homogenates. Therefore, to obtain sufficient protein, ischemic or contralateral MCA from five mice were pooled for measurement of CD45 protein. Anti-Nox2 and anti-eNOS monoclonal antibodies were purchased from BD Biosciences (both 1∶1000), anti-3-nitrotyrosine monoclonal antibody and anti-CD45 polyclonal antibody were from Abcam (both 1∶500). Immunoreactive bands were detected by enhanced chemiluminescence and quantified using a ChemiDoc XRS molecular imager (BioRad). For Nox2, 3-nitrotyrosine and eNOS, immunoreactive bands were normalized to intensity of corresponding bands for β-actin (Cell Signaling; 1∶3000). For CD45 Western blotting, a 5% polyacrylamide gel was used allow adequate protein separation above 150 kDa. As such, there was no separation of proteins <50 kDa in size and therefore measurement of β-actin levels was not possible. Therefore, in these experiments, equal protein loading was confirmed using Ponceau S staining.

### Drugs

Ketamine was purchased from Parnell Laboratories (Australia), L-012 from Wako Pure Chemicals (Japan) and PDB from Calbiochem (USA). U46619 (in methyl acetate) was purchased from Sapphire Biosciences (Australia), xylazine from Troy laboratories (Australia), and all other drugs from Sigma. L-012 and PDB were prepared at 100 mmol/L and 10 mmol/L, respectively, in 100% DMSO and subsequently diluted in Krebs-HEPES solution. U46619 was prepared at 1 mmol/L in 100% EtOH and subsequently diluted in Krebs-bicarbonate solution. All other drugs for myograph experiments were dissolved and diluted in Krebs-bicarbonate solution. For experiments using L-012 and PDB, the final concentration of DMSO was ≤0.2%.

### Data analysis

All results are presented as mean±SEM. Statistical comparisons were performed using one or two-way ANOVA with a Bonferroni multiple comparison post hoc test, log-rank test, one-sample, paired or unpaired *t* test, as appropriate. *P*<0.05 was considered statistically significant.

## Results

### Degree of hypoperfusion, mortality, sensorimotor deficit and infarct volume

**Figure 1:**
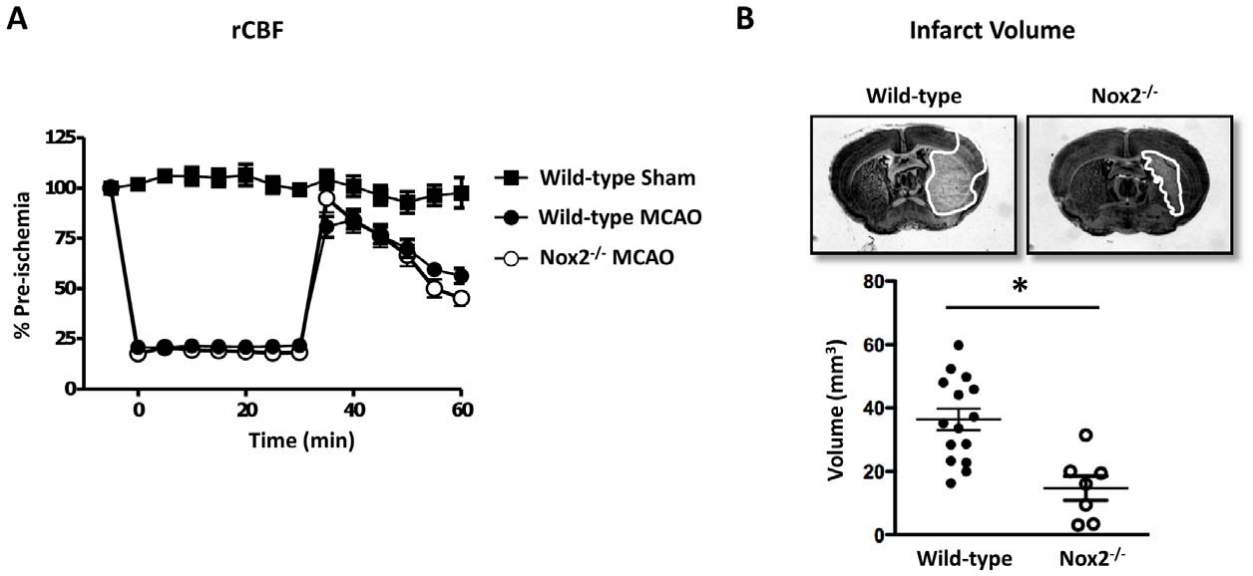
Effect of middle cerebral artery occlusion on cerebral blood flow and infarct volume. Regional cerebral blood flow (rCBF; n = 7 for sham, n = 60 for wild-type and n = 22 for Nox2^-/-^; A) and infarct volumes at 24 h following middle cerebral artery occlusion (MCAO; B) in wild-type and Nox2-deficient (Nox2^-/-^) mice. rCBF was measured during MCAO and during reperfusion (30 min). Representative (n = 15 for wild type and n = 7 for Nox2^-/-^) coronal brain sections are shown for wild-type and Nox2^-/-^ mice at 24 h after MCAO with the infarct area outlined in white (B, top). All results are presented as mean±SEM. *P<0.05 vs. wild-type (unpaired *t* test).

Following insertion of the monofilament in WT or Nox2^-/-^ mice (Fig 1A, n = 60 for WT and n = 22 for Nox2^-/-^) cortical rCBF was reduced by ∼75%, indicative of successful MCA occlusion. Upon withdrawal of the monofilament, flow increased towards 100% indicative of effective reperfusion and then gradually decreased to approximately 50% of pre-ischemic levels after 30 min of reperfusion. In sham-operated WT mice, rCBF remained at ∼100% for the duration of the monitoring period (Fig 1A). Mortality during the 23.5 h reperfusion period was 11/72 (15%) in WT mice and 1/23 (4%) in Nox2^-/-^ mice (log-rank test of survival; *P* = 0.07). Sensorimotor function, as assessed by latency to fall in the hanging wire test, was significantly greater in Nox2^-/-^ mice (37.0±6.7 s, n = 12) than in WT mice (19.8±4.9 s; n = 15, *P*<0.05). In sham-operated WT mice, the latency to fall was 57.3±2.8 s (n = 7). Consistent with the protective effects of Nox2 deletion, Nox2^-/-^ mice had smaller infarct volumes than WT mice (Fig 1B, *P*<0.05, n = 7–15). There were no detectable infarcts in sham-operated WT mice (data not shown). Edema volume also tended to be smaller in Nox2^-/-^ mice (6.6±4.2 mm^3^) compared with WT mice (11.8±2.2 mm^3^; *P* = 0.13).

### Superoxide production by middle cerebral arteries

**Figure 2:**
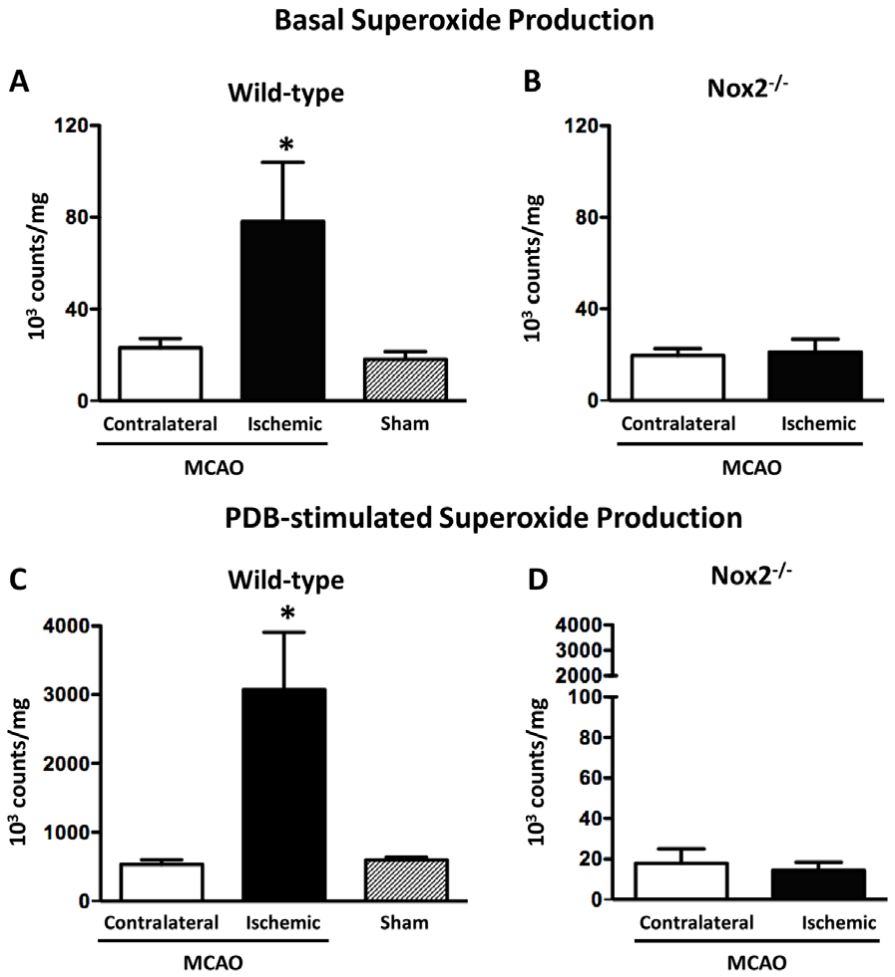
Effect of cerebral ischemia-reperfusion on vascular superoxide production. Basal (A and B) and phorbol-12, 13-dibutyrate (10 µmol/L; PDB: Nox2 activator)-stimulated (C and D) superoxide production by middle cerebral arteries (MCA) as measured by 100 µmol/L L-012-enhanced chemiluminescence. **A.** Contralateral and ischemic MCA from wild-type mice 24 h after MCA occlusion, and pooled left and right MCA from sham-operated wild-type mice. **B.** Contralateral and ischemic MCA from Nox2-deficient (Nox2^-/-^) mice 24 h after MCAO. **C.** Contralateral and ischemic MCA from wild-type mice 24 h after MCA occlusion (MCAO), and pooled left and right MCA from sham operated wild-type mice. **D.** Contralateral and ischemic MCA from Nox2^-/-^ 24 h after MCAO. All results are expressed as 10^3^ counts/mg of dry tissue weight and given as mean±SEM (A and C, n = 9 for both groups of MCAO; n = 7 for sham; B and D, n = 6 for both groups). *P<0.05 vs. contralateral MCA (one way ANOVA with a Bonferroni mutiple comparison post hoc test).

In arteries isolated from WT mice 24 h after MCAO, basal superoxide production by ischemic MCA was ∼3-fold greater than levels generated by contralateral MCA, or by MCA from sham-operated WT mice (Figure 2A; *P*<0.05). By contrast, in ischemic MCA from Nox2^-/-^ mice, basal superoxide production was not elevated compared with contralateral MCA (Fig 2B).

In ischemic MCA from WT mice the Nox2 activator, PDB, profoundly increased superoxide production by ∼50-fold above basal levels (ischemic MCA: basal 52±13 vs. PDB 2784±689 10^3^ counts/mg; *P*<0.05, n = 9, Figs 2A, 2C). PDB-stimulated superoxide levels were ∼6-fold greater in ischemic MCA than in contralateral MCA, and in MCA from sham-operated WT mice (Fig 2C; *P*<0.05 ischemic vs. contralateral and sham). In Nox2^-/-^ vessels after MCAO, PDB did not increase superoxide production above basal levels (ischemic MCA: basal 20±3 vs. PDB 18±7 10^3^ counts/mg, n = 6, Figs 2B, 2D), indicating that PDB selectively increases superoxide by activating Nox2 oxidase.

### Middle cerebral artery nitric oxide function

**Figure 3:**
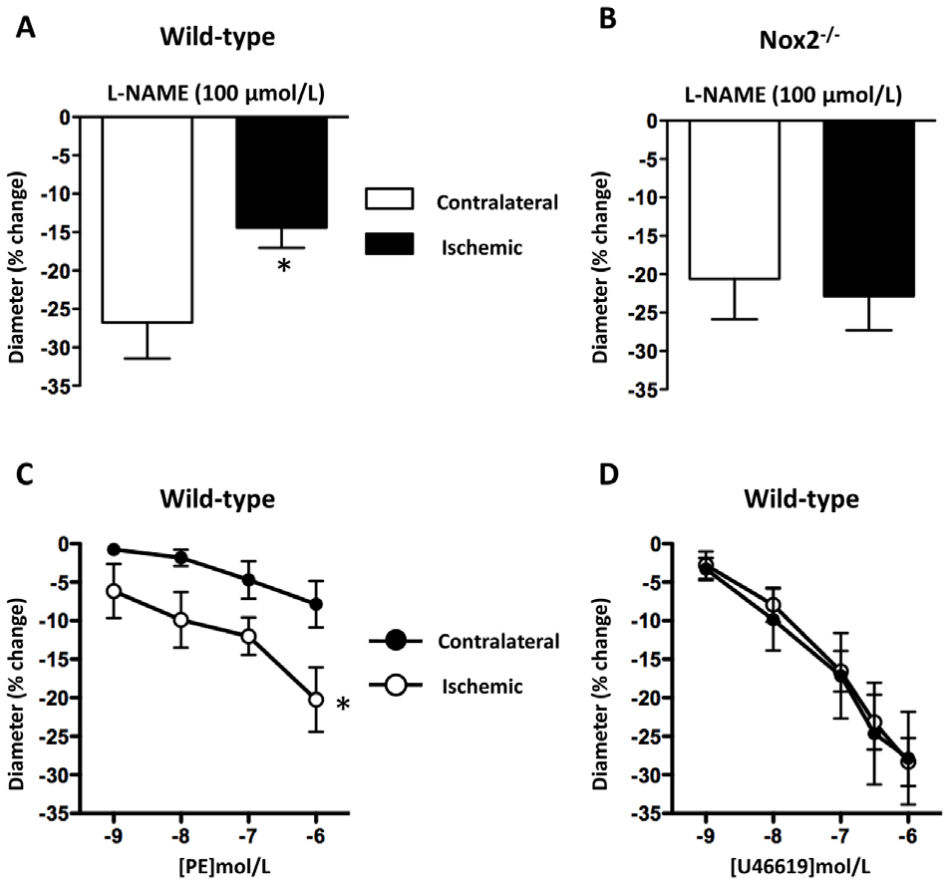
Effect of cerebral ischemia-reperfusion on vascular function. Vasoconstrictor responses of isolated contralateral and ischemic middle cerebral arteries (MCA) from wild-type (A) and Nox2-deficient (Nox2^-/-^; B) mice to L-NAME (100 µmol/L) 24 h after MCA occlusion. Also shown are vasoconstrictor responses of contralateral and ischemic MCA from wild-type mice to phenylephrine (PE: 1 nmol/L-1 µmol/L; C) and U46619 (1 nmol/L-1 µmol/L; D). Results are expressed as% change in intraluminal diameter and given as mean±SEM (A, n = 9 for contralateral, n = 10 for ischemic; B, n = 6 for contralateral, n = 6 for ischemic; C, n = 6 for contralateral, n = 4 for ischemic; D, n = 7 for contralateral, n = 6 for ischemic). *P<0.05 vs. contralateral MCA (A: unpaired *t* test; C: two-way ANOVA with a Bonferroni multiple comparison post-hoc test).

During the equilibration period, MCA did not develop significant myogenic tone. Final baseline diameters of ischemic and contralateral MCA were similar between all groups (WT ischemic, 122±2 µm; WT contralateral, 118±3 µm; Nox2^-/-^ ischemic, 111±8 µm; Nox2^-/-^ contralateral, 114±5 µm). The magnitude of L-NAME-induced constriction of ischemic MCA from WT mice was ∼50% of that in contralateral MCA (Figure 3A, *P*<0.05) indicative of reduced NO function following ischemia-reperfusion. By contrast, in ischemic MCAs from Nox2^-/-^ mice constrictor responses to L-NAME were similar to those in contralateral MCA (Fig 3B). We also found that constrictor responses to phenylephrine were significantly greater in ischemic MCA from WT mice compared with contralateral MCA (Fig 3C, *P*<0.05), also indicative of reduced NO function after ischemia-reperfusion, whereas constrictor responses to U46619 did not differ between ischemic and contralateral MCA (Fig 3D).

### Nox2, 3-nitrotyrosine, eNOS, and CD45 expression

**Figure 4:**
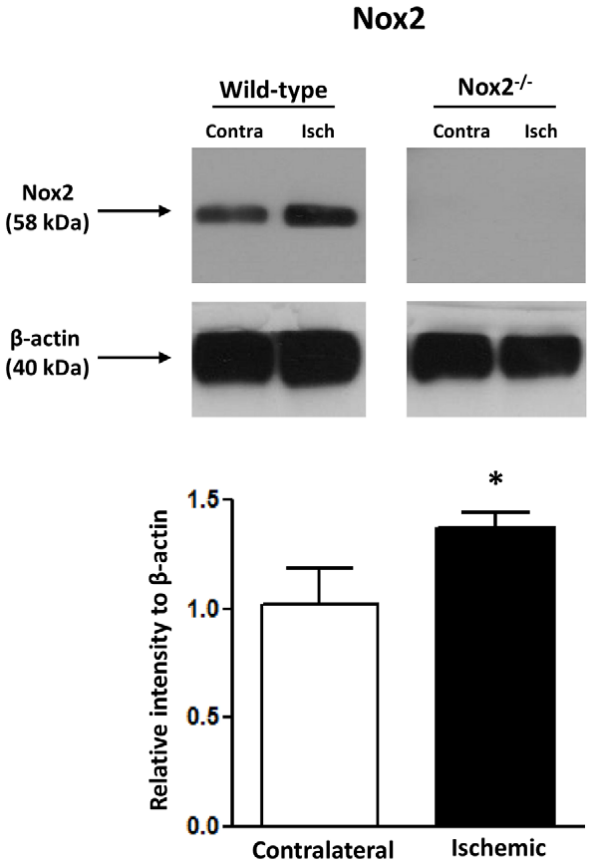
Effect of cerebral ischemia-reperfusion on vascular Nox2 expression. Representative Western blots (top) showing protein expression of Nox2 in contralateral (Contra) and ischemic (Isch) middle cerebral artery homogenates from wild-type and Nox2-deficient (Nox2^-/-^) mice at 24 h after MCA occlusion. Also shown is a summary of immunoreactive band intensity (bottom). Values are expressed as relative intensity normalized to β-actin intensity, and are given as mean±SEM (n = 4 for all groups). *P<0.05 vs. contralateral (one sample *t*-test).

**Figure 5:**
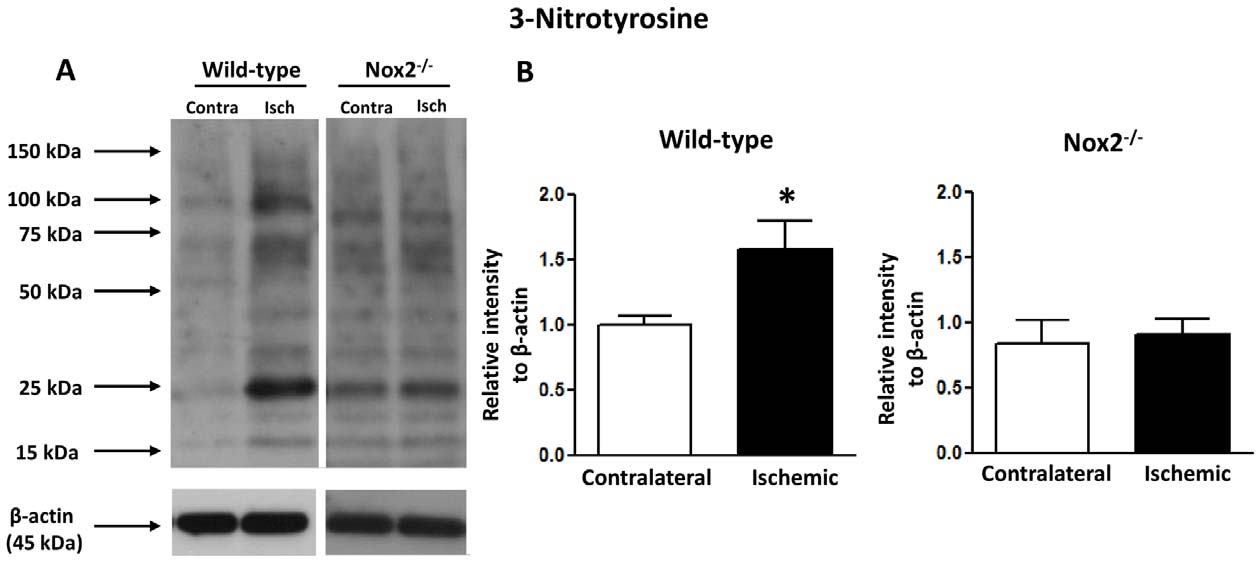
Effect of cerebral ischemia-reperfusion on vascular 3-nitrotyrosine levels. Representative Western blots (A) showing expression of 3-nitrotyrosine in contralateral (Contra) and ischemic (Isch) middle cerebral artery homogenates from wild-type and Nox2-deficient (Nox2^-/-^) mice at 24 h after MCA occlusion. Also shown is a summary of immunoreactive band intensity (bottom). Values are expressed as relative intensity normalized to β-actin intensity, and are given as mean±SEM (n = 5 for all groups). *P<0.05 vs contralateral (one-way ANOVA with a Bonferroni multiple comparison post-hoc test).

**Figure 6:**
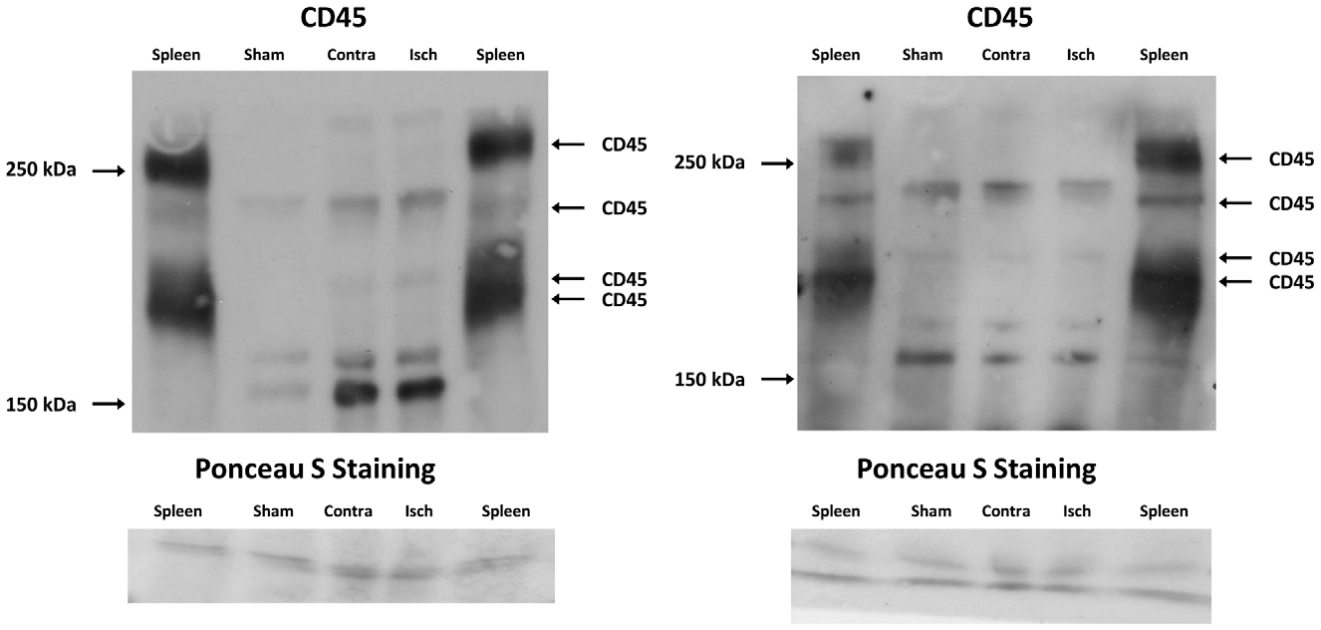
Effect of ischemia-reperfusion on CD45 expression in cerebral arteries. Western blots (n = 2) showing CD45 levels in sham, contralateral (Contra) and ischemic (Isch) middle cerebral artery homogenates from wild-type mice at 24 h after MCA occlusion. Spleen homogenates were used as positive controls. MCA homogenates from 5 mice were pooled in order to obtain sufficient protein levels.

Using homogenates of ischemic and contralateral MCA from Nox2^-/-^ mice subjected to MCAO as a negative control, we found Nox2 to run as a single band at ∼58 kDa in WT MCA (Fig 4). Expression of Nox2 protein was ∼1.4-fold higher in homogenates of ischemic MCA from WT mice than in contralateral MCA (Fig 4, *P*<0.05). In MCA homogenates from WT and Nox2^-/-^ mice following MCAO, 3-nitrotyrosine immunoreactive bands (indicative of peroxynitrite production and protein nitration) were observed between 15 kDa and 150 kDa. Analysis of all bands within this range revealed that in WT mice, 3-nitrotyrosine levels were ∼1.5-fold higher in ischemic than in contralateral MCA (Fig 5A, B). By contrast, in Nox2^-/-^ mice, 3-nitrotyrosine levels were comparable between ischemic and contralateral MCA (Fig 5A, B), and similar to levels in contralateral MCA from WT mice. Protein expression of eNOS was similar between all groups (WT ischemic, 0.99±0.16; WT contralateral, 1.04±0.14; Nox2^-/-^ ischemic, 1.02±0.12; Nox2^-/-^ contralateral, 1.02±0.08 relative expression to β-actin, n = 5 each group, *P*>0.05). CD45 (a pan leukocyte marker) is reported to migrate with apparent molecular weights of 150–250 kDa in mice, depending on the isoform and the extent of glycosylation.[28] Indeed, four CD45 immunoreactive bands were observed between 150–250 kDa in spleen homogenates (Fig 6). In ischemic, contralateral and sham MCA homogenates, immunoreactive bands were observed at approximately 160, 170, 200 and 230 kDa, however only the 200 kDa band corresponded to a band detected in the spleen positive control (Fig 6). The intensity of this band did not appear different between sham, contralateral and ischemic MCA homogenates.

## Discussion

The findings of this study collectively provide the first demonstration that cerebral ischemia-reperfusion induces oxidative/nitrosative stress and endothelial dysfunction via increased expression and activity of Nox2 oxidase in the cerebral artery wall. Firstly, following MCAO in WT mice, superoxide production in the ischemic MCA was selectively and markedly augmented under basal conditions and in response to a Nox2 oxidase activator. Secondly, using an inhibitor of NOS in studies of vascular function, we found evidence that NO bioavailability and/or function was selectively attenuated in the ischemic MCA of WT mice. Thirdly, the oxidative stress and endothelial dysfunction in the ischemic MCA were associated with higher levels of protein tyrosine nitration and of Nox2 protein, the catalytic subunit of an isoform of NADPH oxidase that is highly expressed in the endothelium. Fourthly, we found that none of the changes observed after MCAO in the ischemic artery of WT mice were present in ischemic arteries of Nox2^-/-^ mice. These findings definitively demonstrate that both the activity and expression of Nox2 oxidase are increased in the cerebral circulation following ischemia-reperfusion, leading to ROS-dependent endothelial dysfunction and protein damage.

It is likely that dysregulation of the cerebral circulation compromises perfusion of the post-ischemic brain, and would thus adversely impact stroke outcome by increasing the likelihood of vascular cognitive impairment and secondary stroke. While much research has sought to understand the mechanisms of primary neuronal injury following stroke, relatively little attention has been devoted to the identification of factors that cause cerebrovascular dysfunction. It is known that superoxide levels in the cerebral circulation increase dramatically during the initial stages of post-ischemic reperfusion [7], [9], [10], [11], and that this state of oxidative stress may persist for several days [8]. Nox2 oxidase is one of five isoforms of the NADPH oxidase family of ROS-generating enzymes, and is emerging as a major mediator of oxidative stress and dysfunction in the cerebral circulation during a number of disease states [12], [14], [15], [16], [17]. Nox2 oxidase is primarily expressed in the endothelium of blood vessels and is capable of generating relatively large amounts of superoxide, analogous to the NADPH oxidase in phagocytes responsible for the respiratory burst [12]. We have previously reported that superoxide levels in the cerebral circulation are normally much higher than in a range of systemic arteries [29], [30], which could result in a relatively lower threshold for oxidative toxicity existing in cerebral vessels when superoxide levels are raised above normal during disease. Importantly, when generated in the same biological compartment, superoxide reacts avidly with NO leading to decreased NO bioavailability and the formation of peroxynitrite, a highly reactive and damaging species that causes nitration of tyrosine residues on proteins. Thus, increased activity of Nox2 oxidase in endothelial cells could lead to substantial superoxide production resulting in inactivation of endothelium-derived NO and generation of peroxynitrite.

In the present study we first sought to test whether superoxide production is elevated in the mouse cerebral circulation after ischemia-reperfusion. We found that basal superoxide levels in the ischemic MCA of WT mice were substantially greater (∼3-fold) than in the contralateral MCA or in MCA from sham-operated mice. We also found that superoxide production in the ischemic MCA could be profoundly augmented by a stimulus of Nox2 oxidase (i.e. PDB), and that this effect was much greater than that observed in non-ischemic contralateral arteries. As mentioned, endothelial dysfunction is a major pathological consequence of vascular oxidative stress and occurs in part via the superoxide-dependent interruption of NO signaling. In the setting of post-ischemic reperfusion, diminished NO function has been reported to occur in the cerebral circulation [10], [31], [32], but the role of Nox2 oxidase in such dysfunction has not been examined. To address this question, we next obtained strong functional evidence for impaired NO-mediated function in mouse cerebral arteries after MCAO. Specifically, we found that L-NAME-induced vasoconstriction was selectively attenuated in the ischemic arteries of WT mice. Furthermore, constrictor responses to an α-adrenoceptor agonist (which are typically modulated by endothelium-derived NO [33]) were significantly augmented in those vessels, whereas constrictor responses to a thromboxane A_2_ mimetic were unaffected. Moreover, we found that there were increased levels of 3-nitrotyrosine in ischemic arteries from WT mice, indicative of protein nitration by peroxynitrite and thus inactivation of NO by superoxide.

To directly test whether increased Nox2 oxidase activity underlies these detrimental effects of ischemia-reperfusion on the cerebral artery wall, we performed equivalent MCAO studies in Nox2^-/-^ mice. In contrast to WT mice, we found that basal superoxide levels were not elevated in the ischemic MCA from Nox2^-/-^ mice. Moreover, PDB failed to increase superoxide levels above basal levels in either ischemic or contralateral MCA from Nox2^-/-^ mice, verifying that PDB increases cerebrovascular superoxide production by selectively activating Nox2 oxidase. Consistent with these findings, there were also no abnormalities of constrictor responses to L-NAME, nor were there increased 3-nitrotyrosine levels in ischemic arteries from Nox2^-/-^ mice. Thus, our data provide the first direct and definitive evidence that Nox2 oxidase is the key mediator of augmented superoxide production, endothelial dysfunction and nitrosative stress following cerebral ischemia-reperfusion.

There is good evidence from studies on Nox2 oxidase-deficient mice that elevated activity of this isoform contributes to neuronal injury following ischemic stroke [18], [21], [34], [35], and that the protective effects of the Nox oxidase inhibitor apocynin occur via inhibition of this Nox isoform [19], [20]. More recently, evidence for a role for Nox4 oxidase-derived ROS in neuronal damage has also been reported, whereby genetic deletion or pharmacological inhibition of Nox4 oxidase conferred neuroprotection in permanent and transient models of cerebral ischemia [36]. By contrast, there is divided evidence as to whether Nox1 oxidase activity contributes to stroke damage [36], [37], [38]. Consistent with previous studies of Nox2^-/-^ mice, we found in the present study that infarct volume and neurological impairment were markedly reduced in Nox2^-/-^ versus WT mice. However, our additional findings that the vascular dysfunction that occurs in the cerebral circulation following ischemic stroke is also Nox2-dependent raises the possibility that the preservation of vascular function, in addition to direct neuronal or glial cell protective mechanisms, contributes to the broader beneficial effects of Nox2 oxidase deletion and/or inhibition after ischemic stroke.

To explore a possible molecular basis for augmented Nox2-dependent superoxide levels, we examined the effect of ischemia-reperfusion on cerebral artery Nox2 expression. Using Western blotting, we observed a Nox2 immunoreactive band at approximately 58 kDa in MCA homogenates from ischemic and non-ischemic cerebral hemispheres of WT mice, which was absent in homogenates of MCA from Nox2^-/-^ mice. Analysis of this band revealed that expression levels were ∼1.4-fold higher in ischemic versus non-ischemic arteries. It has been reported that cerebral ischemia-reperfusion is associated with immune cell adhesion and accumulation of inflammatory cells in the cerebral vasculature and brain.[6], [39], [40] Leukocytes express a functionally active Nox2 oxidase [12], and thus, it is conceivable that adherent/infiltrating leukocytes may contribute to the augmented cerebral vascular superoxide production following ischemia-reperfusion. Therefore, to test this we next measured expression levels of the pan leukocyte marker CD45 in MCA following ischemia-reperfusion. Using spleen homogenates as a positive control, we detected one CD45 immunoreactive band in contralateral and ischemic MCA homogenates. However, it did not appear that there were differences in the relative levels of CD45 between contralateral and ischemic MCA. Thus, it appears unlikely that increased leukocyte adherence/infiltration accounts for the Nox2-dependent changes following ischemia-reperfusion, but instead point towards increased vascular cell Nox2 oxidase expression/activity - probably in endothelium - as the main contributing factor. Mechanisms that regulate Nox2 oxidase expression and/or activity in cerebral arteries after stroke remain to be examined. Recently, casein kinase 2 (CK2) was identified as a negative regulator of NADPH oxidase [41]. Interestingly, CK2 activity is rapidly reduced in mouse brains during ischemia-reperfusion, leading to an increase in NADPH oxidase activity and expression of Nox2 [41]. Thus, dysregulation of CK2 may be an important underlying cause of NADPH oxidase-driven neuronal injury in ischemic stroke [41]. If such a relationship also exists between CK2 and Nox2 oxidase in cerebral arteries, it may similarly contribute to elevations in cerebrovascular Nox2 oxidase activity after ischemia-reperfusion. Increased expression of endothelin and angiotensin receptors is reported to occur in the cerebral vasculature after ischemic stroke [2], and indeed stroke is associated with elevated production of pro-inflammatory cytokines, endothelin-1 and angiotensin II [2], [42] – all of which can increase the activity and expression of NADPH oxidases. Thus, it is plausible that several mechanisms may influence Nox2 expression and activity in the cerebral vasculature after stroke.

In summary, we have provided evidence that vascular Nox2 oxidase plays a critical role in elevated superoxide production by cerebral arteries following ischemia-reperfusion. This increase in vascular Nox2 oxidase-derived superoxide appears to cause impaired endothelium-dependent, NO-mediated vasodilator function, most likely via the direct inactivation of NO and the formation of peroxynitrite. The ultimate impact of Nox2 oxidase-mediated endothelial dysfunction on stroke outcome remains to be clarified, however, these studies highlight vascular Nox2 oxidase as a potential novel vaso- and neuro-protective target for stroke therapy.

## References

[1] Fagan SC, Hess DC, Hohnadel EJ, Pollock DM, Ergul A (2004). Targets for vascular protection after acute ischemic stroke.. Stroke.

[2] Edvinsson LI, Povlsen GK (2011). Vascular plasticity in cerebrovascular disorders.. J Cereb Blood Flow Metab.

[3] Gorelick PB, Scuteri A, Black SE, DeCarli C, Greenberg SM (2011). Vascular contributions to cognitive impairment and dementia.. Stroke.

[4] Broughton BR, Reutens DC, Sobey CG (2009). Apoptotic mechanisms after cerebral ischemia.. Stroke.

[5] Woodruff TM, Thundyil J, Tang SC, Sobey CG, Taylor SM (2011). Pathophysiology, treatment, and animal and cellular models of human ischemic stroke.. Mol Neurodegener.

[6] Moskowitz MA, Lo EH, Iadecola C (2010). The science of stroke: mechanisms in search of treatments.. Neuron.

[7] Kontos CD, Wei EP, Williams JI, Kontos HA, Povlishock JT (1992). Cytochemical detection of superoxide in cerebral inflammation and ischemia in vivo.. Am J Physiol Heart Circ Physiol.

[8] Miller AA, Dusting GJ, Roulston CL, Sobey CG (2006). NADPH-oxidase activity is elevated in penumbral and non-ischemic cerebral arteries following stroke.. Brain Res.

[9] Nelson CW, Wei EP, Povlishock JT, Kontos HA, Moskowitz MA (1992). Oxygen radicals in cerebral ischemia.. Am J Physiol Heart Circ Physiol.

[10] Xie H, Ray PE, Short BL (2005). NF-_K_B activation plays a role in superoxide-mediated cerebral endothelial dysfunction after hypoxia/reoxygenation.. Stroke.

[11] Yemisci M, Gursoy-Ozdemir Y, Vural A, Can A, Topalkara K (2009). Pericyte contraction induced by oxidative-nitrative stress impairs capillary reflow despite successful opening of an occluded cerebral artery.. Nat Med.

[12] Drummond GR, Selemidis S, Griendling KK, Sobey CG (2011). Combating oxidative stress in vascular disease: NADPH oxidases as therapeutic targets.. Nat Rev Drug Discov.

[13] Miller AA, Drummond GR, Sobey CG (2006). Novel isoforms of NADPH-oxidase in cerebral vascular control.. Pharmacol Ther.

[14] De Silva TM, Broughton BRS, Drummond GR, Sobey CG, Miller AA (2009). Gender influences cerebral vascular responses to angiotensin II through Nox2-derived reactive oxygen species.. Stroke.

[15] Girouard H, Park L, Anrather J, Zhou P, Iadecola C (2006). Angiotensin II attenuates endothelium-dependent responses in the cerebral microcirculation through Nox-2-derived radicals.. Arterioscler Thromb Vasc Biol.

[16] Miller AA, De Silva TM, Judkins CP, Diep H, Drummond GR (2010). Augmented superoxide production by Nox2-containing NADPH oxidase causes cerebral artery dysfunction during hypercholesterolemia.. Stroke.

[17] Park L, Anrather J, Girouard H, Zhou P, Iadecola C (2007). Nox2-derived reactive oxygen species mediate neurovascular dysregulation in the aging mouse brain.. J Cereb Blood Flow Metab.

[18] Brait VH, Jackman KA, Walduck AK, Selemidis S, Diep H (2010). Mechanisms contributing to cerebral infarct size after stroke: gender, reperfusion, T lymphocytes, and Nox2-derived superoxide.. J Cereb Blood Flow Metab.

[19] Chen H, Song YS, Chan PH (2009). Inhibition of NADPH oxidase is neuroprotective after ischemia-reperfusion.. J Cereb Blood Flow Metab.

[20] Jackman KA, Miller AA, De Silva TM, Crack PJ, Drummond GR (2009). Reduction of cerebral infarct volume by apocynin requires pretreatment and is absent in Nox2-deficient mice.. Br J Pharmacol.

[21] Kahles T, Luedike P, Endres M, Galla H-J, Steinmetz H (2007). NADPH oxidase plays a central role in blood-brain barrier damage in experimental stroke.. Stroke.

[22] Pollock JD, Williams DA, Gifford MA, Li LL, Du X (1995). Mouse model of X-linked chronic granulomatous disease, an inherited defect in phagocyte superoxide production.. Nat Genet.

[23] Jackman KA, Miller AA, Drummond GR, Sobey CG (2009). Importance of NOX1 for angiotensin II-induced cerebrovascular superoxide production and cortical infarct volume following ischemic stroke.. Brain Res.

[24] Judkins CP, Diep H, Broughton BR, Mast AE, Hooker EU (2010). Direct evidence of a role for Nox2 in superoxide production, reduced nitric oxide bioavailability and early atherosclerotic plaque formation in ApoE-/- mice.. Am J Physiol Heart Circ Physiol.

[25] Bullen ML, Miller AA, Dharmarajah J, Drummond GR, Sobey CG (2011). Vasorelaxant and antiaggregatory actions of the nitroxyl donor isopropylamine NONOate are maintained in hypercholesterolemia.. American Journal of Physiology - Heart and Circulatory Physiology.

[26] Marrelli SP, Khorovets A, Johnson TD, Childres WF, Bryan RM (1999). P2 purinoceptor-mediated dilations in the rat middle cerebral artery after ischemia-reperfusion.. Am J Physiol Heart Circ Physiol.

[27] Miller AA, Drummond GR, Mast AE, Schmidt HHHW, Sobey CG (2007). Effect of gender on NADPH-oxidase activity, expression, and function in the cerebral circulation: role of estrogen.. Stroke.

[28] Thomas ML (1989). The leukocyte common antigen family.. Annual Review of Immunology.

[29] Miller AA, Drummond GR, Schmidt HH, Sobey CG (2005). NADPH oxidase activity and function are profoundly greater in cerebral versus systemic arteries.. Circ Res.

[30] Miller AA, Drummond GR, De Silva TM, Mast AE, Hickey H (2009). NADPH oxidase activity is higher in cerebral versus systemic arteries of four animal species: role of Nox2.. Am J Physiol Heart Circ Physiol.

[31] Cipolla MJ, Smith J, Kohlmeyer MM, Godfrey JA (2009). SKCa and IKCa Channels, myogenic tone, and vasodilator responses in middle cerebral arteries and parenchymal arterioles: effect of ischemia and reperfusion.. Stroke.

[32] Mayhan WG, Amundsen SM, Faraci FM, Heistad DD (1988). Responses of cerebral arteries after ischemia and reperfusion in cats.. Am J Physiol Heart Circ Physiol.

[33] Budzyn K, Marley PD, Sobey CG (2004). Chronic mevastatin modulates receptor-dependent vascular contraction in eNOS-deficient mice.. Am J Physiol Regul Integr Comp Physiol.

[34] Kunz A, Anrather J, Zhou P, Orio M, Iadecola C (2006). Cyclooxygenase-2 does not contribute to postischemic production of reactive oxygen species.. J Cereb Blood Flow Metab.

[35] Walder CE, Green SP, Darbonne WC, Mathias J, Rae J (1997). Ischemic stroke injury is reduced in mice lacking a functional NADPH oxidase.. Stroke.

[36] Kleinschnitz C, Grund H, Wingler K, Armitage ME, Jones E (2010). Post-Stroke Inhibition of Induced NADPH Oxidase Type 4 Prevents Oxidative Stress and Neurodegeneration.. PLoS Biol.

[37] Jackman KA, Miller AA, Drummond GR, Sobey CG (2009). Importance of NOX1 for angiotensin II-induced cerebrovascular superoxide production and cortical infarct volume following ischemic stroke.. Brain Res.

[38] Kahles T, Kohnen A, Heumueller S, Rappert A, Bechmann I (2010). NADPH oxidase Nox1 contributes to ischemic injury in experimental stroke in mice.. Neurobiology of Disease.

[39] Miyazaki T, Kimura Y, Ohata H, Hashimoto T, Shibata K (2011). Distinct effects of tissue-type plasminogen activator and SMTP-7 on cerebrovascular inflammation following thrombolytic reperfusion.. Stroke.

[40] Ishikawa M, Stokes KY, Zhang JH, Nanda A, Granger DN (2004). Cerebral microvascular responses to hypercholesterolemia: roles of NADPH oxidase and P-selectin.. Circ Res.

[41] Kim GS, Jung JE, Niizuma K, Chan PH (2009). CK2 Is a novel negative regulator of NADPH oxidase and a neuroprotectant in mice after cerebral ischemia.. J Neurosci.

[42] Tuttolomondo A, Di Raimondo D, di Sciacca R, Pinto A, Licata G (2008). Inflammatory cytokines in acute ischemic stroke.. Curr Pharm Des.

